# Editorial: 100 years of electrophysiology in psychiatry: clinical diagnostics, prediction and therapy

**DOI:** 10.3389/fpsyt.2025.1708100

**Published:** 2025-10-06

**Authors:** Mehmet Kemal Arikan

**Affiliations:** ^1^ Department of Biostatistics, Faculty of Medicine, Uskudar University, Istanbul, Türkiye; ^2^ Kemal Arikan Psychiatry Clinic, Istanbul, Türkiye

**Keywords:** EEG, ERP, microstate, TMS, substance usage disorders (SUDs), gambling disorder (GD), major depression (MDD), Hans Berger

In 1924, Hans Berger, a psychiatrist from Jena, Germany, recorded the first human electroencephalogram (EEG). His discovery, initially met with skepticism, soon became one of the most enduring achievements in neuroscience and clinical medicine. One hundred years later, EEG remains central in both neurology and psychiatry. This centennial is not merely a historical commemoration; it is an opportunity to reflect on the evolution of EEG, its present role in psychiatry, and its future as a digital biomarker platform.

Following Berger’s breakthrough, EEG spread rapidly across Europe and North America. By the 1930s, Alfred Loomis and colleagues in the United States had identified distinct sleep stages, demonstrating the EEG’s relevance to physiology and psychiatry (1). In 1935-1936, Pauline and Hallowell Davis first studied the sensory evoked potential (2), and later Grey Walter pioneered cognitive event-related potential (ERPs), laying the groundwork for cognitive electrophysiology (3). Over the decades, EEG has proved invaluable in epilepsy, sleep medicine, cognitive neuroscience, and eventually psychiatry, where its potential as a biomarker continues to expand.

The progress of EEG has always been international, shaped by diverse contributions. Pioneering figures such as Turan Itil advanced pharmaco-EEG, demonstrating that drug effects could be systematically evaluated through cerebral electrical patterns (4). Ismet Karacan became internationally recognized for his work in sleep EEG, which included landmark studies on depression-related sleep disturbances (5). Erol Başar reframed oscillatory activity as the building blocks of cognition and emotion, bringing rhythms such as gamma to the forefront of psychiatric neuroscience (6, 7). These contributions, alongside others worldwide, highlight EEG’s enduring adaptability.

Although interest once seemed to shift toward novel neuroimaging methods such as MRI and PET, EEG has continued to maintain and even strengthen its position. Bibliometric analyses confirm exponential growth in EEG publications in recent years across conditions from ADHD to dementia (8–10). This persistence reflects EEG’s unique strengths: millisecond temporal resolution, non-invasiveness, and adaptability to emerging digital and computational tools. Moreover, advances in digitalization, large-scale data storage, and machine learning have transformed EEG from a descriptive tool into a predictive platform for biomarkers and clinical interventions.

The contributions included in this Research Topic illustrate these strengths and signal future directions for psychiatric EEG research. Each paper demonstrates how EEG is being used to bridge clinical applications, methodological innovation, and the development of digital biomarkers.

Balconi et al. propose integrating EEG biomarkers into the neurocognitive screening of executive functions in substance and behavioral addictions. They emphasize that alterations in reward sensitivity and executive control, hallmarks of both substance use disorder and gambling disorder, can be reliably assessed using oscillatory and ERP markers. Their perspective suggests that EEG could support not only diagnosis but also rehabilitation and relapse prevention in addiction.

İlhan and Arıkan examine the impact of repetitive and deep transcranial magnetic stimulation (rTMS and dTMS) on quantitative EEG in patients with depression. Their findings underscore EEG’s potential to capture neural changes induced by brain stimulation and highlight the correlation between EEG and symptom improvement. This work exemplifies how EEG can serve as a translational bridge between neurostimulation methods and clinical psychiatry.

Özcoban and Tan focus on major depressive disorder (MDD), analyzing absolute and relative power alongside asymmetry indices. Their results add to the growing literature on oscillatory markers of depression, pointing toward EEG’s capacity to differentiate between clinical states and potentially predict prognosis. Such work demonstrates that EEG biomarkers can complement traditional clinical assessments, enhancing objectivity in psychiatric diagnostics.

Shiroma et al. validate a novel sheet-type portable EEG device (HARU-1) by replicating Berger’s original alpha rhythm finding, the so-called “Berger effect.” Demonstrating that a portable system can reliably detect this foundational EEG signature is more than a symbolic achievement—it marks a step toward ecological neuroscience. Portable EEG may allow clinicians to monitor patients in real-world settings, tracking mood fluctuations, relapse risk, and treatment effects outside the laboratory.

Perrottelli et al. provide a systematic review of ERP microstates in psychiatric disorders. Moving beyond single ERP peaks, microstate analysis captures large-scale spatiotemporal brain dynamics. Their review shows consistent alterations in schizophrenia and mood disorders, highlighting microstates as promising biomarkers for psychiatric research and offering a framework to study temporal brain function in clinical populations.

Taken together, these five contributions showcase the vitality and breadth of EEG applications in psychiatry today. From addiction and depression to stimulation studies, portable devices, and methodological advances, EEG proves itself to be both versatile and resilient. Far from being overshadowed by neuroimaging, EEG continues to offer unique insights that no other method can match.

Looking ahead, two directions seem particularly promising. First, portable and wearable EEG devices will expand our ability to monitor brain function beyond the clinic, enabling continuous, real-world measurement of cognitive and emotional states. Such ecological biomarkers may guide personalized interventions and reshape psychiatric care. Second, oscillatory markers such as gamma rhythms remain at the forefront as indicators of psychiatric and neurologic disorders (7). Their integration with modern computational approaches may help define EEG’s role in precision psychiatry.

As we honor Berger’s centennial, it is fitting to recognize the role of scientific societies in sustaining EEG’s progress. The Clinical EEG & Neuroscience Society (ECNS) exemplifies this tradition, fostering collaboration across psychiatry, neurology, and neuroscience. These networks remind us that EEG’s strength lies in its international community of researchers and clinicians.

Hans Berger could not have imagined the global reach his discovery would one day achieve. Yet a century later, EEG remains a cornerstone of psychiatric neuroscience—adaptable, digital, and poised for the future. The studies featured in this Research Topic confirm that EEG is thriving, evolving, and prepared to shape the next century as a digital biomarker platform. We invite readers to explore these contributions, engage with the possibilities they represent, and continue the shared effort of illuminating the living brain.
